# Network Identification of Hormonal Regulation

**DOI:** 10.1371/journal.pone.0096284

**Published:** 2014-05-22

**Authors:** Daniel J. Vis, Johan A. Westerhuis, Huub C. J. Hoefsloot, Ferdinand Roelfsema, Jan van der Greef, Margriet M. W. B. Hendriks, Age K. Smilde

**Affiliations:** 1 Department of Metabolic and Endocrine Diseases, University Medical Center Utrecht, Utrecht, The Netherlands; 2 Biosystems Data Analysis, Swammerdam Institute for Life Sciences, University of Amsterdam, Amsterdam, The Netherlands; 3 Netherlands Metabolomics Centre, Leiden, The Netherlands; 4 Department of Endocrinology and Metabolic Diseases, Leiden University Medical Center, Leiden, The Netherlands; 5 TNO Quality of Life, Zeist, The Netherlands; Umeå University, Sweden

## Abstract

Relations among hormone serum concentrations are complex and depend on various factors, including gender, age, body mass index, diurnal rhythms and secretion stochastics. Therefore, endocrine deviations from healthy homeostasis are not easily detected or understood. A generic method is presented for detecting regulatory relations between hormones. This is demonstrated with a cohort of obese women, who underwent blood sampling at 10 minute intervals for 24-hours. The cohort was treated with bromocriptine in an attempt to clarify how hormone relations change by treatment. The detected regulatory relations are summarized in a network graph and treatment-induced changes in the relations are determined. The proposed method identifies many relations, including well-known ones. Ultimately, the method provides ways to improve the description and understanding of normal hormonal relations and deviations caused by disease or treatment.

## Introduction

Serum hormone concentrations vary considerably, both between subjects and within subjects, between days and during the 24-h day-night cycle. The variation in serum concentration is the result of secretion from endocrine glands into the circulation and clearance from the blood [1]. Secretion consists of basal (nonpulsatile) and pulsatile (burstlike) secretion. The serum concentration profile is hormone-specific [1], [2]. It is increasingly recognized that the pulsatile hormone secretion process supports important biological functions and that a more constant blood hormone concentration tends to diminish the sensitivity of the target tissues to that particular hormone [3].

The awareness of the variability in hormone concentrations emerged in the 1960's with the introduction of radioimmunoassays. The appreciation of within-day variability is still not implemented in the current clinical evaluation of a patient's endocrine status, except the diagnosis of Cushing's disease [4]. Generally, patients are tested at a single time point in the morning under fasting conditions. Such diagnostic tests ignore the variability and important pulsatile features of circulating hormone concentrations. In rare cases patients are tested at one or few time points after experimental perturbation (for example, the oral glucose tolerance test [5], or the GHRH-arginine stimulation test [6]).

Hormone secretion is regulated by other hormones; their dynamic interrelations modulate critical functions in target tissues. For example, insulin increases the glucose uptake by the liver and muscle [7]. On the other hand, hormone secretion is often influenced by several other factors, including gender, body composition, age, and other hormones [8], [9].

The regulation of pituitary hormone secretion is controlled by hypothalamic hormones, delivered via the pituitary portal system and feedback signals from the periphery acting on the different pituitary cell types and/or hypothalamic nuclei, synthesizing and secreting pituitary-stimulating or inhibiting neurohormones or transmitters. Feedback signals include hormones synthesized by endocrine glands, for example, estrogens, testosterone, thyroid hormones, cortisol, and IGF-I, and metabolic signals, including leptin and insulin. The knowledge of the complex central processing of the feedback signals (either positive or negative) is largely based on physiological studies performed in animals. While other information about the human signalling is derived from studies in patients with activating or muting gene deletions, or by clamping studies in which one or more signals are fixed [10]–[13]).

One way to characterize the regulatory relations is to construct networks of these interrelations. Network representations of dynamic patterns can be obtained by models like dynamic Bayesian networks or Hidden Markov Models (HMM) [14]–[16]. In HMM, networks can be built from time-delayed associations. The applicability of HMM for detecting the interrelation between hormones is limited for at least two reasons. First, many hormone interdependencies are reciprocal and are best represented by a cyclical graph, which has to be accounted for in the more complex Hierarchical Hidden Markov Models. In addition, the interrelation delays cannot be assumed to be equal among all hormone relations, which prohibits the use of (first order) HMM. Although higher order Markov Models do allow for unequal delays, many additional parameters need to be estimated which makes the model complex and more difficult to interpret. Hence, the existing HMM methods are too rigid for application to hormonal systems. Therefore, a new strategy with less rigid assumptions was adopted. The proposed network inference methodology is capable of handling some types of reciprocity and unequal delays.

The methodology is illustrated with a case study in obese but non-diabetic women. The detected relations between circulating hormones before and after treatment are analyzed and visualized in a network. Relating secretion patterns of circulating hormones attempts to unravel known and unknown relations between hormone systems. The latter is without pretending that any unknown relation, if statistically significant, is proof of a direct relation. However, such (unexpected) relations may motivate further investigations in human or animal models to describe detailed mechanistic dependencies. The study used to illustrate this methodology investigated the acute effects of bromocriptine on leptin levels, while keeping caloric intake constant. Leptin is the satiety hormone that signals the volume of adipose store to the brain. Bromocriptine is reported to lower the plasma leptin concentration in subjects with prolactinoma, without affecting body weight [17]. Because of the implicated metabolic processes, prolactin, GH, TSH, glucose and insulin are also measured, as are the HPA hormones ACTH and cortisol.

## Materials and Methods

### 2.1 Experimental data

The data that is used to illustrate the network inference method comes from a clinical study involving a premenopausal cohort (n = 18) of obese individuals (BMI>30 *kg*/*m* ^2^) with mild insulin resistance [17]–[19]. The average height of the subjects was 1.68 *cm* (range 1.55–1.76), with a SEM of 1.2 *cm*. The average weight of the subjects was 94.4 *kg* (range 83.7–118.1), with a SEM of 2.51 *kg*.

The ethical board of Leiden University Medical Center evaluated and approved the (original) study. All subjects provided their written consent. The subjects were recruited through advertisements in local newspapers [17]. Exclusion criteria were acute or chronic disease, depression, head trauma, smoking, alcohol abuse, recent transmeridian flights, nightshift work, and recent blood donation. All participants were required to have regular menstrual cycles, the studies were done in the early follicular phase of the menstrual cycle [17]. A total of 246 ml of blood was collected during each occasion. The subjects were instructed to remain recumbent, except for bathroom visits. The subjects were provided with a standardized liquid breakfast (0930), lunch (1300), and dinner (1830). The caloric content was fixed at 2100 kcal/d, of which 35% came from fat, 49% from carbohydrates and 16% from proteins [17]. The lights were switched off at 2300, and back on at 0730.

**Figure 1:**
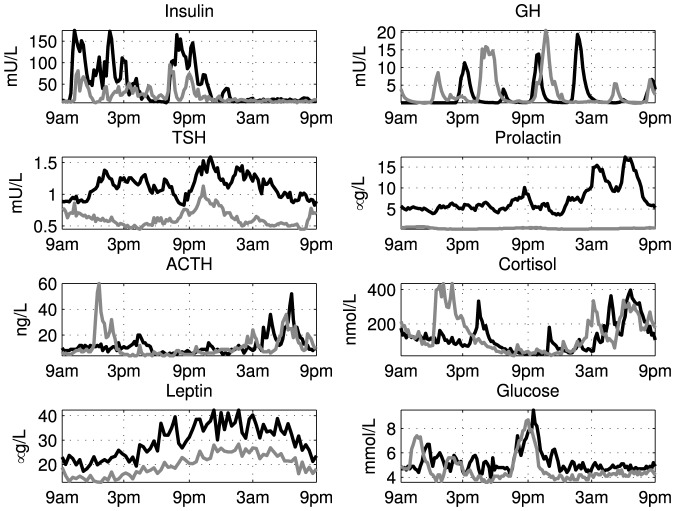
Twenty four-hour hormone concentration profiles in a representative healthy obese female subject who was treated with bromocriptine for eight days. The black lines show the concentrations before treatment, the gray lines after treatment. Bromocriptine caused a profound decrease in prolactin levels, and a decrease in serum TSH, ACTH and leptin concentrations, while increasing GH levels. In addition, insulin and glucose levels also were deminished by bromocriptine.

Blood samples were collected every 10 minutes for 24 hours to assess the endocrine state of the women. The samples were drawn from the antecubital vein, in which a canula and a stopcock was kept patent by continuous NaCl and heparin infusion [17]. In each sample the concentration of ACTH, cortisol, GH, TSH, prolactin, leptin, insulin, and glucose was determined. This list is referred to as the hormones, even though it is noted that glucose is not a hormone, this convention is for brevity only. The subjects were subsequently treated with two daily doses of 2.5 mg bromocriptine during 8 days. On the last day of the treatment the subjects underwent again a 24-h blood sampling study. Figure 1 shows the 24-h hormone concentration profiles in a representative subject.

### 2.2 Methods

A network representation offers a comprehensive view of the dynamic relations that exist between hormones. The network summarizes the complex regulatory patterns into interpretable descriptions. The network inference strategy uses estimated secretion rates to assess the association between hormones.

#### 2.2.1 Pulse identification

The estimation of secretion pulses from observational data is not trivial. The secretion and decay are difficult to delineate and the translation to mathematical formulae of these mechanisms does not yield a well-posed problem. A constraint based method with a minimal set of assumptions was used to estimate the mass and timing of secretion pulses [1]. Recently two other operator-independent deconvolution programs for simultaneously estimating hormone pulses, secretory mass and hormone half-lives were described (AutoDecon [20] and an algorithm by Keenan et al [21], [22]) but here we use our own method (VisPulse [1]). As the overlap between AutoDecon and VisPulse estimates is considerable [1], highly similar results are to be expected when one of the other methods was to be used.

The use of secretion profiles, assessed by pulse identification methods, was motivated by the desire to reduce the auto-correlation present in the original concentration profiles, see also Vis et al [23]. Such auto-correlation distorts many types of measures, including cross correlation. Besides reducing the auto-correlation, the use of secretion profiles also focuses the analysis to the hormone release into the serum compartment.

#### 2.2.2 Hormone association

As a measure of the association between two hormones the lagged Pearson product moment correlation (that is, cross correlation) is used on the secretion pulses. It is assumed that the lag of highest positive and/or lowest negative (optimal) cross-correlation between two hormones represents the time domain in which regulation between two hormones is effectuated. This characteristic is used to create a network representation; if a significant optimal cross-correlation exists, it is stated that there is a relationship between hormones. The unit of the lag is the time interval between two sampling points, 10 minutes, and is referred to by the Greek letter *τ*.

### 2.3 Network inference

#### 2.3.1 Inference of a static network

A static or instantaneous network is based on links between hormones that are present without a time lag. This is the commonest application of association networks found in the literature. The reason for that is largely based on the lack of time series data. The associations are usually made with a number of subjects presumed to be in a steady-state. Since this is a time series of a set of subjects time lags can be included. As a reference, also a static network analysis is performed.

A static network is constructed by associating the pulse pattern of one hormone with the pulse pattern of another. The links of a network are included when the resultant association is significant. The association values are calculated per subject, these values are deemed significant when they are similar across the set of subjects, as assessed by a *t*-test. A false discovery rate correction is applied and a threshold of 5% is used.

A large body of research discusses the merits of conditioning on other variables to limit the detection of indirect relations. The procedure is better known as partialization, the variation of other variables is regressed out of the variable pair of interest before calculating the association in the first pair. This procedure is widely used in graphical Gaussian network modeling [24], [25]. We applied such partialization on the static network by calculating the partial correlation for each pair per subject (for details: see the Appendix). The partial network is included to let the reader inspect the relative gain in interpretability achieved with this procedure for this data. The correlation and partial correlation networks are given in the Results section.

#### 2.3.2 Dynamic network inference

Some relations between two hormones are characterized by a delay between them. The dynamic network appreciates such a delay, while the static network does not. The relation between the hormones is characterized by the cross-correlation. The optima, or extremes, in the cross-correlation function define the delay. To allow for both positive and negative delayed relations, two optima are defined for each pair of hormones. The time window in which regulatory relations are considered is four hours. The mathematical details of the inference can be found in the Appendix.

After calculating the optima, three validation steps are included. First, to test the robustness of the found optima a resampling strategy was followed. This is performed by repeatedly leaving out two subjects and redoing the analysis. The modes of all the calculated cross-correlation functions are then examined to assess the robustness (see Appendix). Secondly, the significance of the cross-correlation value at the optimal delay is assessed by testing the average cross-correlation at the optimum against the assumption that there is no relation, that is, the association is zero. The test is one sided because the optimum is either positive or negative. The resulting *p*-values are subjected to a false discovery rate (FDR) correction at the *α* = 0.01 level. The explicit optimization of searching for the optimal delay is compensated for by adopting a more conservative threshold. Note that this sort of optimization is not used in the static network inference, nor the evaluation of the treatment effect (see later). Thirdly, it is assessed whether the optimal delay is different from zero by using the between individual variation of the cohort. In case of a significant difference there is evidence (but not proof) of a causal relation between the two hormones, which is shown as an arrow in the network. The delayed relations are reported such that when *b* follows *a* the network shows an arrow between the two, for example, *a*→*b*. More details about the mathematics involved in these procedures is shown in the Appendix.

The rationale for setting up this analysis is to detect changes in the regulatory relations between hormones. Treatments or different phenotypic states can influence the relation between two hormones. Changes in the regulatory relation between two hormones can be twofold; their delay can change and/or the intensity of the regulation at a specific delay can change. In this paper we select a reference situation with a specific (optimal) delay calculated for this situation. All other situations are then compared to the reference situation in terms of intensity of the regulation at the pre-selected reference delay. This captures both facets of change: i) if the optimum delay shifts in the new situation, then the intensity at the (then non-optimal) pre-selected delay tends to diminish and ii) if the optimal delay remains the same but the tightness of the regulations changes, then this will manifest itself as an intensity change.

In the preceding section on static network inference the idea of partialization was introduced. In a dynamic network partialization also has relevance, but it is not clear how to perform this. Whereas in the static network the partial correlation of two hormones has to be established while correcting for all other six hormones, this number of partializing variables explodes when considering time delays. How many delays should be included the partialization step? Obviously, such an explosion of possibilities leaves room for chance results. Methods to validate the resulting partial correlations are far from trivial. Hence, we restrict ourselves to correlations instead of partial correlations for the dynamic nets. Moreover, use of hormone secretion values lowers the risk of finding indirect correlations considerably.

#### 2.3.3 Treatment effect

The study used two sampling series of subjects, one before and one after a treatment with bromocriptine. To assess whether the treatment did change the amplitude in the network, the before treatment and after treatment cross correlation values were evaluated using a paired t-test (see Equation 11). The treatment effect is assessed and subjected to an FDR correction. Effects are deemed significant, after FDR correction, at *α* = 0.05, additionally, the relations with a tendency (0.05<*α*<0.10) to a treatment effect are also reported.

## Results

### 3.1 Pulse identification

**Figure 2:**
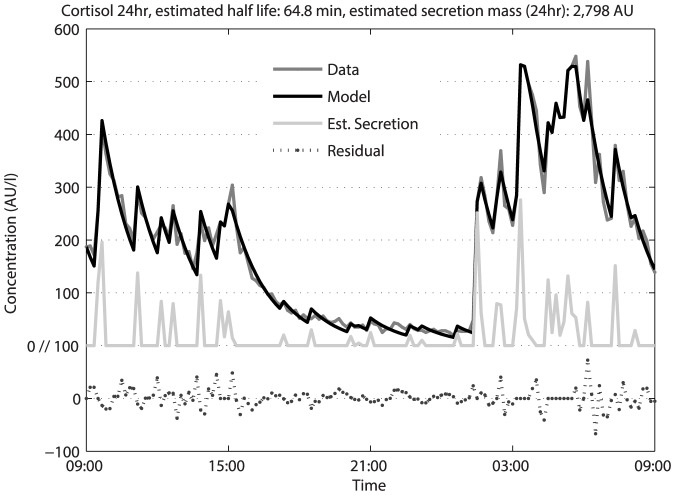
Twentyfour-hour cortisol concentration profile in a healthy obese woman. The secretion amplitude and timing were estimated by VisPulse which is a mathematical model with minimal assumptions, only an exponential decay is assumed and the secretion is assumed to be episodic. The latter is interpreted to mean that only a limited set of time points are involved in the episodic secretion, no assumptions are made about the distribution of the secretion. The residuals are without significant autocorrelation.

Pulsatility is a characteristic of the secretion process of many hormones. An attempt is made to estimate the timing and amplitudes of the secretion pulses with a previously developed numerical method (VisPulse [1]). Figure 2 shows a typical example of a 24-hr hormone series, here an example of cortisol, that was modeled with VisPulse. In gray the measured data is shown and in black the modeled data. The concentration profile shows a clear diurnal pattern with a nadir in the afternoon and evening, and a zenith in the early morning, which is the normal pattern for cortisol. The model correctly identifies the concentration spikes, or pulses, that are characteristic for the 24-hr levels of many hormones, including cortisol. The estimated secretion gives insight in the timing and the mass of the pulses of cortisol released to the general circulation. The data and the minimal model assumptions are well matched as both are in concordance. As discussed in the Method section, all following analyses will be performed on the estimated secretion profile.

### 3.2 Static network inference

The static association network is created by calculating for each subject the correlation between all hormone pairs. For each hormone pair the estimated correlation for all subjects is tested for being unequal to zero by means of a *t*-test. The hormone pair correlation that is significant across subjects is included in the network.

**Figure 3:**
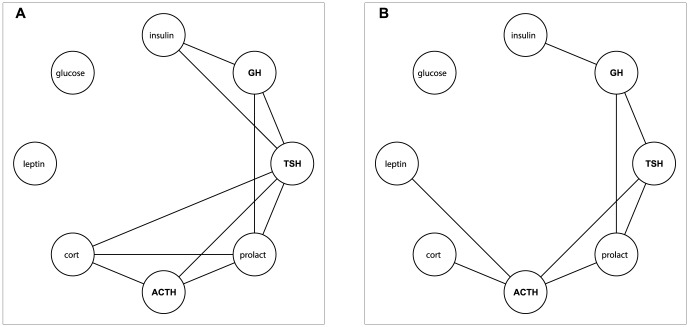
Correlation A and partial correlation B hormone networks. The significant links show a high degree of overlap between the correlation and the partial correlation networks.

Figure 3 shows the results of the analysis on the network. The left panel shows the normal association network, and the right panel shows the partial correlation network. Between the two methods, the majority of links are identical though some difference in the strength is observed. The difference between the normal and the partial network is exemplified by the link between cortisol and TSH, as cortisol is largely regulated by ACTH. The association network identifies a link between cortisol and TSH, while in the partial network the variation in cortisol (driven by ACTH) is removed and identifies no relation between the two. A similar observation is made between cortisol and prolactin.

### 3.3 Dynamic network inference

The network is based on the dynamic interactions between hormones as described by the cross correlation function as documented in the Appendix. To provide a better understanding of the relations between estimated secretion sequences, some examples are highlighted, and the intermediate steps are shown in graphical form. In all results, the before treatment volunteer cohort was used as the reference cohort (see above).

**Figure 4:**
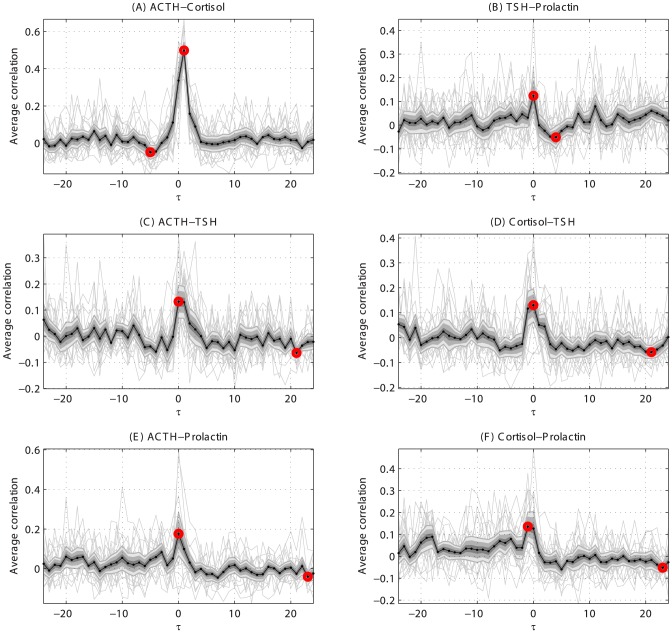
The triplet ACTH, TSH, and prolactin is fully connected in the network. These relation, and the relation with cortisol, are shown in more detail. ACTH-cortisol is clearly connected. The TSH-prolactin relations is more modest but still significant. The relation ACTH-TSH is significant, but shows a wide optimum, interestingly Cortisol-TSH shows a similarly wide optimum which is shifted to the right. ACTH-Prolactin shows a narrow optimum while cortisol-prolactin is again wider and shifted to the right.

First, the relation between hypothalamus-pituitary-adrenal (HPA) axis hormones ACTH and cortisol is shown. Figure 4(A) shows the individual cross correlation profiles on the estimated secretion profile of ACTH and cortisol. The individual results reveal a consistent optimum at lag 1 for all individuals and some heterogeneity in the higher lag ranges. The former translates to a significant relation at lag 1 and the latter to non-significant average cross correlation for higher lags. The algorithm identified the positive and negative optima, but the correlation is significantly different from zero in only the positive optimum. A lag of 1 means that there is a delay of the 10 minutes in the second series compared to the first time series, which is the interval between two sampling points. On the other hand, a lag of 0 means that there is no delay, while a lag of −1 means that the second series precedes the first series.

Second, the significant relation between the two other pituitary hormones prolactin (PRL) and TSH is shown and serves as an example of how the network behaves in cases that are less evident. Figure 4(B) shows the individual relation profiles based on the estimated secretion sequences of prolactin and TSH. The results indicate that some of the secretion events found in prolactin and TSH are coupled, albeit to a significantly lower degree than found for ACTH and cortisol. The negative optimum follows the positive optimum by 30 minutes and may indicate a refractionary period (a temporary inhibition of new events) in both hormones or in the mechanism that drives the both secretion events. For reference, Figure S1 shows these profiles after treatment and Figure S2 shows these profiles for a lean cohort (BMI 21.8 *kg*/*m* ^2^, SEM 0.4, 5 male, 4 female).

With the detailed discussion of the relations between ACTH, cortisol, TSH, and prolactin, an attempt is made to familiarize the reader with the application of the methodological concepts introduced here by showing the crosscorrelation results in full before abstracting these concepts in a network. The idea of directionality will be illustrated here. When the crosscorrelation is highest at lag 0 the two hormones are maximally associated without a lag. When the highest crosscorrelation is not at lag 0, a delay between the two hormones (may) exists. Since not all peaks are sharp ambiguity can arise. For example, when the crosscorrelation at lag 0 and at lag 1 is similar it is not clear whether there is a temporal direction. The directionality is here validated by differentiating the association at the optimal lag against the association at lag 0.

Figures 4(A) and 4(B) illustrate how the method summarizes a relation between hormones and how the detection of optima performs in real data. Having described the detected relations between ACTH-cortisol and TSH-prolactin, it is only natural to wonder whether there are detectable crossed relations between these hormones. Figure 4(C) shows the relation between ACTH-TSH is significant at lag 0 although the association at lag 1 is almost as high. Figure 4(E) points out that the relation between ACTH-prolactin is similar in timing but with some narrower optimum. Since ACTH and cortisol are related (see Figure 4(A)) the relations with TSH on the one hand and prolactin on the other hand are also investigated. Figure 4(D) shows that there is a significant relation between cortisol and TSH, which is not surprising given that ACTH and TSH are significantly associated. The relation between cortisol and prolactin, shown in Figure 4(F), is consistent with the latter relation. It is interesting to note that ACTH-TSH shows an optimum at lag 0 or 1 but that cortisol-TSH shown an optimum at lag −1 or 0, neither relations are with detectable directionality. The relation ACTH-prolactin is sharp and located at lag 0 while the cortisol-prolactin relation is identified at lag −1.

The relations described in the preceding paragraph show the interconnectedness of the hormones which is illustrative of the network structure governing these processes. Figure 4 is the basis for a small part of the network. The positive association between ACTH and cortisol at lag 1 is significant in both the association and the direction. The latter translates to a directed relation between ACTH and cortisol in the network. The negative association is not significant, and thus not included in the network. All other associations are included as described before with the addition that negative relations are shown as dashed lines to allow for easy visual discrimination between positively and negatively associations. Finally, a solid black circle is added to each relation that changed as a result of the treatment. The mathematical details of the procedure are in the Appendix.

**Figure 5:**
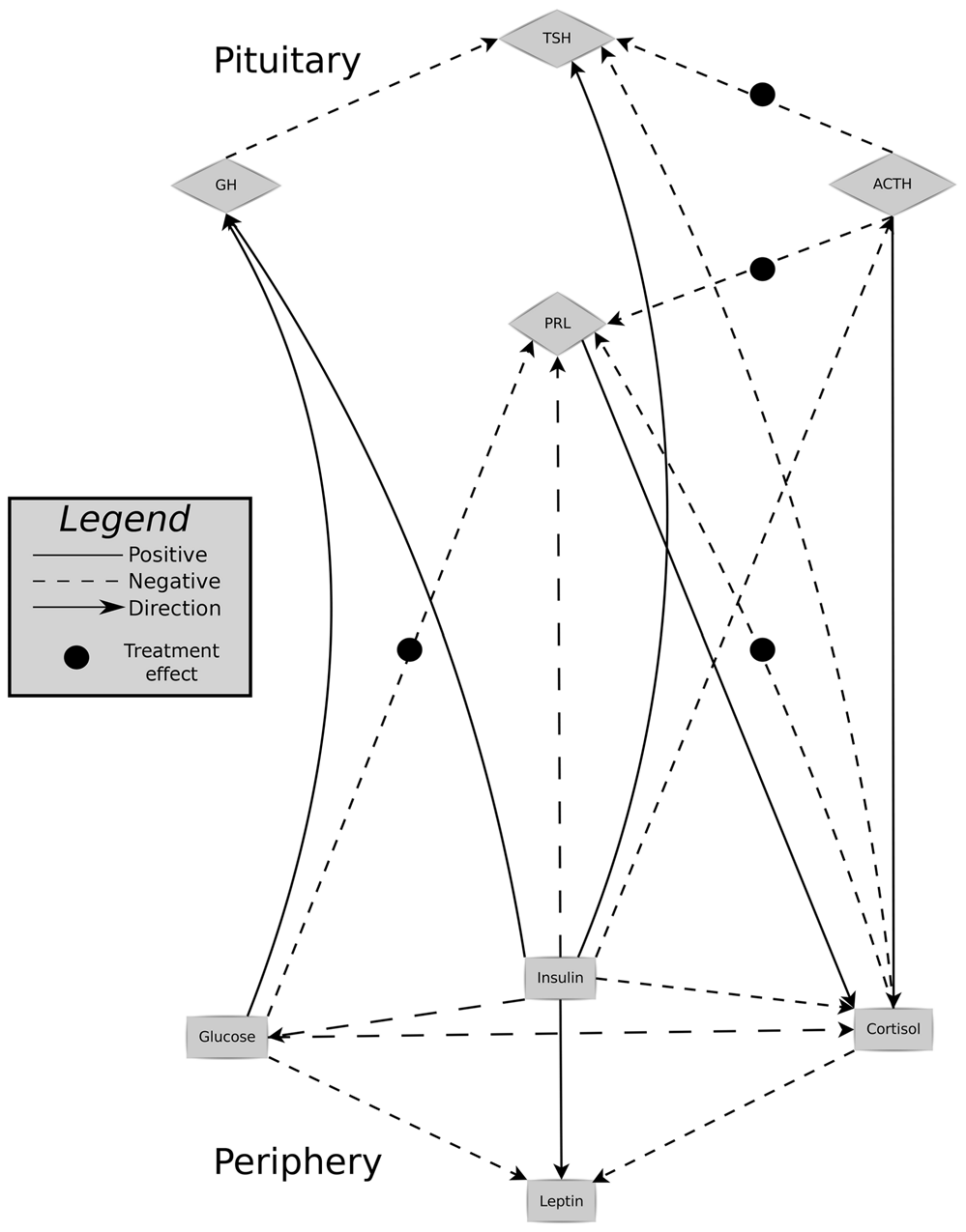
The dynamic hormone network and the treatment effect. Links with an arrow indicate directionality, links without are suppressed. The black circles indicate a significant treatment effect. The top four hormones are release in the pituitary, while the others are released in the periphery, and glucose is ingested as food.

The network with lagged relations is shown in Figure 5. In this figure only the non-instantaneous relations are shown for brevity, the complete list of identified significant relations, directionality, and treatment effects is tabulated in Table 1. Of the 36 identified relations, 8 are significantly affected by the treatment with bromocriptine (and 6 show a tendency to an effect with a *q*-value between 0.05 and 0.10). The majority of the treatment affected relations are targeting either TSH or PRL, but relations that target ACTH, cortisol, insulin and GH are also found.

**Table 1:** The network is based on the data presented in this table.

Relation	Lag in *τ*	Type	Relation q-value	p-value directionality	Treatment effect q-value
GH-INS	6	−	5.432e-07	0.636	0.080∼
INS-GH	15	+	5.064e-03	0.000	0.991
TSH-GH	0	+	9.897e-04	NaN	0.892
PRL-GH	0	+	5.785e-03	NaN	0.012*
GLC-GH	5	−	1.883e-03	0.252	0.366
INS-TSH	15	+	9.897e-04	0.000	0.658
INS-TSH	1	−	3.619e-04	0.091	0.573
GH-TSH	0	+	9.897e-04	NaN	0.892
GH-TSH	10	−	1.397e-03	0.000	0.080∼
PRL-TSH	0	+	1.312e-03	NaN	0.592
ACTH-TSH	0	+	9.897e-04	NaN	0.185
ACTH-TSH	21	−	5.248e-05	0.000	0.043*
CRT-TSH	0	+	1.312e-03	NaN	0.080∼
CRT-TSH	21	−	7.605e-04	0.000	0.051∼
GLC-TSH	1	−	9.562e-03	0.303	0.146
INS-PRL	12	−	7.622e-06	0.000	0.099∼
GH-PRL	0	+	5.785e-03	NaN	0.012*
TSH-PRL	0	+	1.312e-03	NaN	0.592
ACTH-PRL	0	+	4.296e-03	NaN	0.146
ACTH-PRL	23	−	3.854e-03	0.001	0.043*
CRT-PRL	23	−	5.597e-04	0.000	0.007*
GLC-PRL	21	−	1.312e-03	0.005	0.043*
INS-ACTH	6	−	1.196e-03	0.125	0.158
GH-ACTH	3	−	5.069e-03	0.151	0.040*
TSH-ACTH	0	+	9.897e-04	NaN	0.185
PRL-ACTH	0	+	4.296e-03	NaN	0.146
INS-CRT	17	−	2.510e-04	0.005	0.185
PRL-CRT	1	+	1.290e-03	0.817	0.146
ACTH-CRT	1	+	2.109e-11	0.001	0.676
GLC-CRT	2	−	2.682e-03	0.008	0.070∼
INS-LPT	23	+	5.069e-03	0.040	0.687
CRT-LPT	2	−	2.878e-03	0.022	0.185
GLC-LPT	14	−	3.880e-03	0.094	0.146
INS-GLC	1	+	5.085e-03	0.744	0.862
INS-GLC	11	−	1.859e-03	0.003	0.676
GH-GLC	20	+	5.209e-03	0.013	0.291

For each relation the lag, in terms of *τ*, is shown. The type of the relation, positive or negative, is indicated by + or −. The q-value of the identified relations are false discovery rate corrected (at *α* = 0.01). For each relation the p-value for directionality is shown (*H*_0_ : *τ* = 0). The last column shows the q-value of the treatment effect (at *α* = 0.10) and the asterisks (*) marks the significant treatment effect (<0.05), a tilde (∼) a tendency to an effect (0.05<*q*<0.10). (*τ* refers to a 10 minute interval).

## Discussion

The results point out the hormones that appear to be associated during a normal day, and which of these relations changed as a result of the treatment with dopamine d2 agonist bromocriptine. Well-established associations were found for ACTH and cortisol [26]–[29], and between cortisol and TSH [30], [31]. The latter serves as a partial validation of the method. The network further revealed that insulin is with a positive relation to leptin, glucose and GH, and with a negative relation to ACTH, PRL and glucose. These resuls appear inline with literature. It is worthwhile to note that the treatment did not change the relation between insulin and PRL (see Table 1). Similarly, a stable relation is identified between insulin and glucose.

Contrasting the previous examples, the relation between TSH and prolactin is only known to exist under extra-physiological conditions in which exogenous thyrotropin-releasing hormone (TRH) is injected in a non-physiological dose which results in an increase in both TRH and prolactin concentration [32]. Whether the mode of action of TRH is similar under physiological conditions is still unknown. Here we provide evidence that can support the hypothesis that this mechanism is also active under physiological conditions. Based on the relations shown in Figure 4 it is speculated that the associations presented are the result of ACTH directly, ACTH in combination with TRH, or between CRH and TRH.

Previous observations in obese subjects have suggested that central dopamine receptor functioning is impaired [33]. Different studies have demonstrated that short-term treatment with bromocriptine decreased leptin, insulin, TSH, ACTH and PRL secretion [34]. The difference between the network before and after bromocriptine administration might be explained by improved insulin sensitivity. If insulin is important to feedback signaling of different hypothalamic-pituitary hormone systems, gastric bypass surgery in the obese patient might be a suitable model of insulin sensitivity, because of its effect on insulin secretion and sensitivity [35]–[37].

With respect to the experimental data, the largest unknown is the day-to-day intra-subject variation. The latter issue cannot be solved statistically, but requires repeated 24-hr observations of the same individuals, preferably on consecutive days. Due to restrictions on the allowable volume of blood extraction, such is not possible without further advancement in measurement techniques that allow for an order of magnitude reduction in the required sample volume. The latter is notwithstanding plausible additional (ethical) restrictions related to a >48-hr observation-sampling regime.

The present approach of relating secretion patterns of circulating hormones attempts to unravel known and unknown relations between hormone systems. The results are presented without pretending that any unknown relation, if statistically significant, is proof of a direct relation. We have shown how relations and differences can be detected in a case-control type of context by assessing the treatment effect of bromocriptine. The latter idea can be extended to other cases, and normal ranges can be defined for well-defined subgroups. Given such a reference the deviations in the hormone relations can interpreted as a departure from a healthier state [38], [39].

## Conclusions

Detecting relations in systems with a level of complexity that is similar to the human endocrine ensemble is not easy. Our approach gives associations between hormones, and even in cases of detected directionality, no claims about causation can be made.

Nevertheless with the use of observational data we are able to detect many known relations between hormones. This serves as a validation of the method. Most relations are previously described in literature, but not in conjunction with the other relations. In addition to the expected relations we were able to identify some relations, such as TSH and PRL, that are without evidence under physiological conditions.

What our approach adds is the unsupervised detection of these relations in a cohort and the subsequent evalution of a treatment effect. The network approach provides an convenient visualization of the identified relations. The analysis of the intervention with bromocriptine reveals that many of the relations are conserved, pointing to a remarkable consistency in the complex relations between endocrine entities. As the insulin resistance improved as a result of the treatment, the relations involving TSH, cortisol and GH appear the most affected along with the drastic changes in relations with PRL.

## Supporting Information

Materials S1 **Mathematical Appendix and Figures S1 and S2.** **Figure S1. The after treatment relations between ACTH, TSH, prolactin, and cortisol are shown here in more detail.** ACTH-cortisol is clearly connected. The TSH-prolactin relation is more modest but still significant. The relation ACTH-TSH is significant, but shows a wide optimum, interestingly Cortisol-TSH shows a similarly wide optimum which is shifted to the right. ACTH-Prolactin shows a narrow optimum while cortisol-prolactin is again wider and shifted to the right. **Figure S2. The lean control relations between ACTH, TSH, prolactin, and cortisol are shown here in more detail.** ACTH-cortisol is clearly connected. The TSH-prolactin relation is more modest but still significant. The relation ACTH-TSH is significant, but shows a wide optimum, interestingly Cortisol-TSH shows a similarly wide optimum which is shifted to the right. ACTH-Prolactin shows a narrow optimum while cortisol-prolactin is again wider and shifted to the right.
(PDF)
