# Supplementary material for: Network Identification of Hormonal Regulation
Source: PLoS One. 2014 May 22;9(5):e96284. doi: 10.1371/journal.pone.0096284 (PMC4031081; doi:10.1371/journal.pone.0096284)
Supplement: Materials S1 — Mathematical Appendix and Figures S1 and S2. Figure S1. The after treatment relations between ACTH, TSH, prolactin, and cortisol are shown here in more detail. ACTH-cortisol is clearly connected. The TSH-prolactin relation is more modest but still significant. The relation ACTH-TSH is significant, but shows a wide optimum, interestingly Cortisol-TSH shows a similarly wide optimum which is shifted to the right. ACTH-Prolactin shows a narrow optimum while cortisol-prolactin is again wider and shifted to the right. Figure S2. The lean control relations between ACTH, TSH, prolactin, and cortisol are shown here in more detail. ACTH-cortisol is clearly connected. The TSH-prolactin relation is more modest but still significant. The relation ACTH-TSH is significant, but shows a wide optimum, interestingly Cortisol-TSH shows a similarly wide optimum which is shifted to the right. ACTH-Prolactin shows a narrow optimum while cortisol-prolactin is again wider and shifted to the right. (PDF) [file pone.0096284.s001.pdf]

Network Identification of Hormonal Regulation

Daniel J. Vis[1,2,3,*] Johan A. Westerhuis[2,3] Huub C. J. Hoefsloot[2,3] Ferdinand Roelfsema[4] Jan van der Greef[3,5] Margriet M. W. B. Hendriks[1,3,‡] Age K. Smilde[2,3,‡]
1 Department of Metabolic and Endocrine Diseases, University Medical Center Utrecht, The Netherlands
2 Biosystems Data Analysis, Swammerdam Institute for Life Sciences, University of Amsterdam, The Netherlands
3 Netherlands Metabolomics Centre, Leiden, The Netherlands
4 Department of Endocrinology and Metabolic Diseases, Leiden University Medical Center, The Netherlands
5 TNO Quality of Life, Zeist, The Netherlands
‡ Equal contributors
∗ E-mail: science@danielvis.nl

S1 Appendix

This appendix provides an overview of the methods and the statistics that are used.

S1.1 Data

Eighteen obese premenopausal subjects in this study were sampled for 24-hr at 10-minutes intervals (145 blood samples) [1–3]. The measured hormone concentrations are coded (Equation 1), in which i is the subject ($\{1 \cdots 18\}$), j is the hormone ($\{1 \cdots 8\}$), k is the time index ($\{1 \cdots 145\}$), and o is the measurement occasion (before-after, $\{1, 2\}$). As stated in the Materials and Methods the observed variables in this analysis are seven hormones and glucose, for simplicity they are collectively referred to as hormones.

$$x_{i,j,k,o} \tag{1}$$

S1.2 Pulse estimation

The serum concentration profiles of most hormones display episodic secretion that can be estimated. The model used to estimate the secretion events is described by the following equation [4].

$$x_{i,j,k,o} = x_{i,j,(k-1),o}e^{-\kappa_{i,j,o}} + \phi_{i,j,k,o} + u_{i,j,k,o} \tag{2}$$

In this equation, $x_{i,j,k,o}$ is the hormone concentration as described, $e^{-\kappa}$ is the (estimated) decay rate, $\phi_{i,j,k,o}$ is the (estimated) secretion, and $u_{i,j,k,o}$ is the residual (error) term. The estimated secretion is the basis on which further analysis is performed and the networks were inferred.

S1.3 Metrics

The metrics use are the ordinary Pearson product moment correlation and the lagged Pearson product moment correlation. The former is commonly referred to as correlation while the latter is better known as the cross correlation. As correlation statistics are skewed Gaussian the Fisher-Z transform, the area hyperbolic tangent (*atanh*), is used. After this transform the average can be calculated and the result is back transformed to ordinary correlation space by taking the inverse of the function, the hyperbolic tangent, which then becomes the aggregated cohort statistic. The average Fisher-Z transformed correlation and the standard error of the mean is used to calculate t-values. See, Equations 1-9.

S1.4 Static network metrics

The static metrics are based on the Pearson product moment correlation and are calculated on the estimated secretion profiles. The correlation estimates are Fisher-Z transposed to improve the asymptotic consistency of the estimates.

$$c_{i,j,j',o,0} = atanh(cor(\phi_{i,j,k,o}, \phi_{i,j',k,o})), \tag{3}$$

S1.4.1 Partialization

For the partial correlation the variation of all other variables is regressed out of the $\phi_{i,j,k,o}$ and $\phi_{i,j',k,o}$ pair and the obtained residuals are correlated per individual [5]. No other changes to the algorithm are made.

S1.5 Optima selection

The optima are defined to be extremes in the averaged cross-correlation. To get some understanding of the variability in extremes a resampling scheme was formulated that performed a leave two (individuals) out resampling. The resampling is performed by randomly selecting $n-2$ out of the n subjects, after which the optima calculation is repeated on that subset. This chain of actions is repeated a 1000 times. The modes of *mic* and *mac* are the robust optima, the selected optima are referred to by e. The lags at which the minima and the maxima cross correlation was found are tabulated, and the 'optimal' τ was defined to be the mode of the entire set of τ. The $\bar{c}_{j,j',o,\tau}$ optima were defined in Equation 4 and 5.

The optima are defined as the values of the averaged cross-correlation.

$$mic_{j,j',o} = argMin_{\tau}(\bar{c}_{j,j',o,\tau}) \tag{4}$$
$$mac_{j,j',o} = argMax_{\tau}(\bar{c}_{j,j',o,\tau}) \tag{5}$$

S1.6 Dynamic network metrics

The cross-correlation analysis is applied to individual subjects. The application of the Fisher Z transformed cross-correlation function [6] is shown in Equation 6, in which i is a particular subject,j and j' are hormones ($j \neq j'$), τ the lag, N the number of time points ($N = 145$), o a particular measurement occasion, and w the total number of lags to consider. In the analysis the upper value of w was set to 24, which is equivalent to 4 hours (6 samples/hour).

$$c_{i,j,j',o,\tau} = atanh(cor(\phi_{i,j,k,o}, \phi_{i,j',k+\tau,o})), (-w \leq \tau \leq w) \tag{6}$$

The aggregation of the individual cross-correlation values is performed as shown in Equation 7, in which: n is the number of subjects ($n = 18$). Equations 8 and 9 show how the standard deviation and t-value are calculated. Since the optima are signed, that is, we search for the highest or lowest values, all tests using this data are single sided.

$$\bar{c}_{j,j',o,\tau} = \frac{1}{n}\sum_{i=1}^{n} c_{i,j,j',o,\tau} \tag{7}$$

$$sc_{j,j',o,\tau} = \sqrt{\frac{1}{n-1}\sum_{i=1}^{n}(c_{i,j,j',o,\tau} - \bar{c}_{j,j',o,\tau})^2} \tag{8}$$

$$tc_{j,j',o,\tau} = \frac{\bar{c}_{j,j',o,\tau}}{\frac{1}{\sqrt{n}} sc_{j,j',o,\tau}} \tag{9}$$

S1.6.1 False discovery rate control

A false discovery rate (FDR) correction is used to control the number of false positives. Controlling the FDR at 5% means that on average 5% of the significant features are truly null (that is, not significant). The Benjamini and Hochsberg procedure [7] was used to control this rate. The default significance level was set at $\alpha = 0.05$. Since the dynamic network is based on optimized values, the optima, a convervative level of $\alpha = 0.01$ was used for this inference. For the treatment effect the tendency ($0.05 < \alpha < 0.10$) of an effect is also reported.

S1.7 Directionality

A nonzero optimal lag estimated from the aggregated cross-correlations implies directionality, which, as effects are believed to emerge after the cause, could imply regulation. The mathematics of these conceptual chains are rooted in theory developed by Fisher [8], Granger [9], Pearl [10, 11] and Dawid [12, 13]. Because the hormone concentrations cannot be assumed to be autoregressive (AR), the standard tests for Granger causality cannot be applied, and a more generic test is used instead.

The null hypothesis is that there is no direction, that is, the correlation values at $\tau = 0$ are the same as the correlation values at optimal τ. Directionality is said to exist when the optimal lag is with higher correlation values than $\tau = 0$.

$$H_0 : E(c_{j,j',o,\tau=0}) = E(c_{j,j',o,e}) \tag{10}$$

The directionality statistics are provided in Table 1. Note that a positive lag indicates that the second variable follows the first with some lag, for negative lags the ordering is reversed.

S1.8 Treatment effect

The optimum lag was estimated in the before treatment situation. The treatment effect is defined as a difference between the correlation values between before and after treatment. In Equation 11, before/after is indicated by 1, 2, e is the optimum, the null hypothesis (Equation 11) is evaluated using a paired t-test.

$$H_0 : E(c_{j,j',1,e}) = E(c_{j,j',2,e}) \tag{11}$$

S1.9 Software

The calculations were performed in Mathworks MATLAB 2011b, except for the calculation of the FDR q-values which was performed in R 2.13.1 using the stats package.

S2 Supplemental material

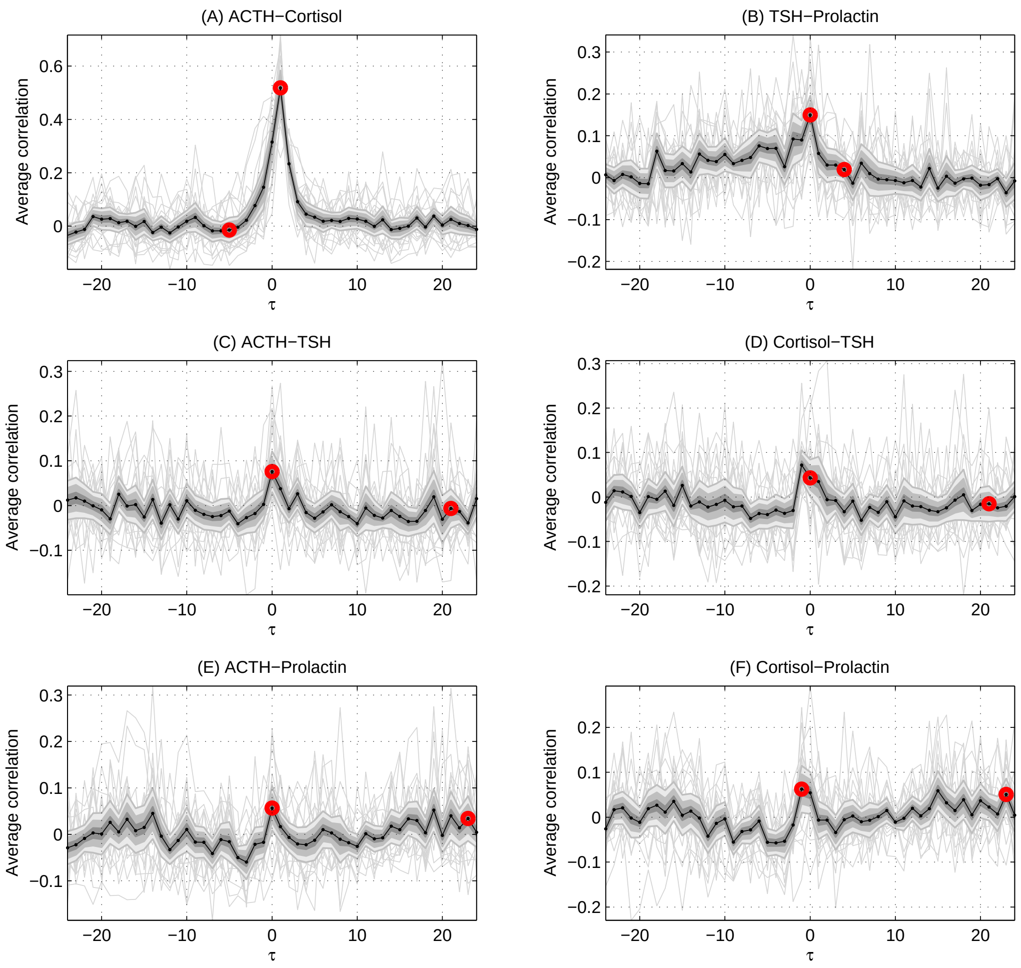

Figure S1: The after treatment relations between ACTH, TSH, prolactin, and cortisol are shown here in more detail. ACTH-cortisol is clearly connected. The TSH-prolactin relation is more modest but still significant. The relation ACTH-TSH is significant, but shows a wide optimum, interestingly Cortisol-TSH shows a similarly wide optimum which is shifted to the right. ACTH-Prolactin shows a narrow optimum while cortisol-prolactin is again wider and shifted to the right.

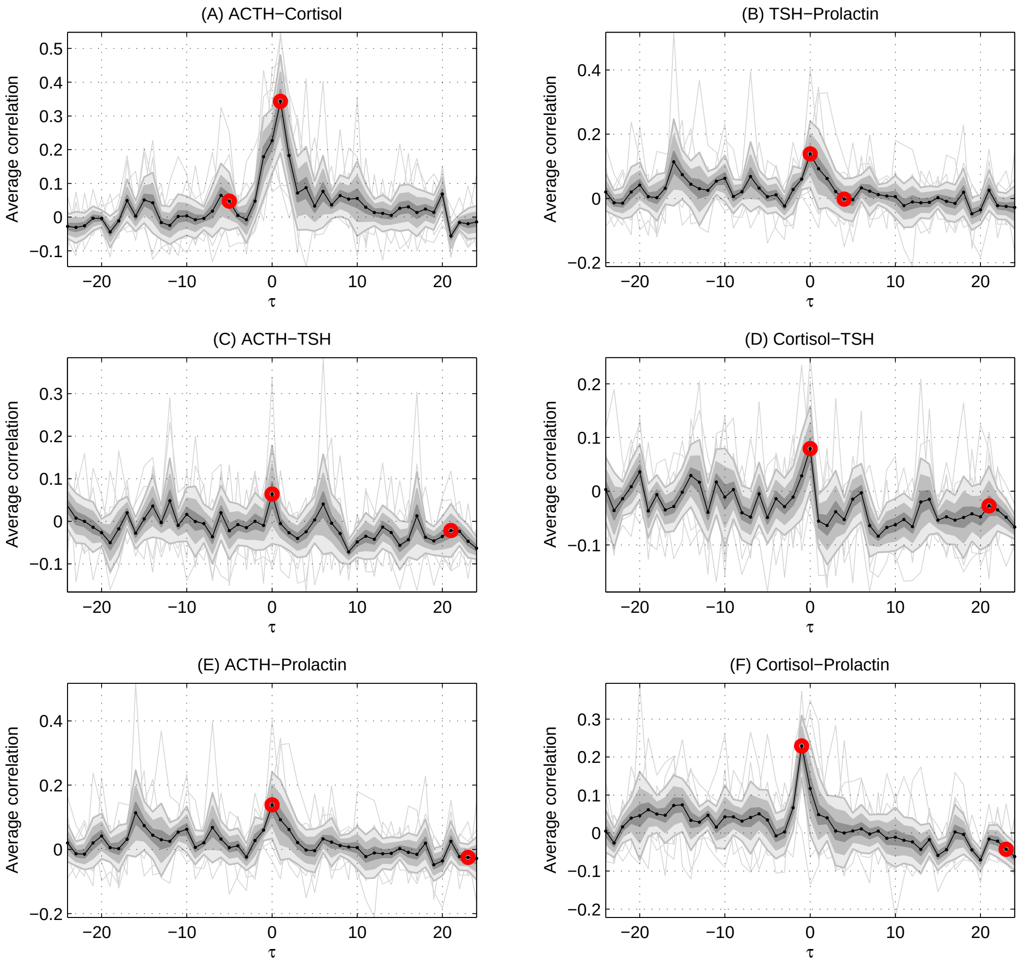

Figure S2: The lean control relations between ACTH, TSH, prolactin, and cortisol are shown here in more detail. ACTH-cortisol is clearly connected. The TSH-prolactin relation is more modest but still significant. The relation ACTH-TSH is significant, but shows a wide optimum, interestingly Cortisol-TSH shows a similarly wide optimum which is shifted to the right. ACTH-Prolactin shows a narrow optimum while cortisol-prolactin is again wider and shifted to the right.

References

[1] Kok P, Roelfsema F, Frölich M, van Pelt J, Meinders AE, et al. (2008) Short term treatment with bromocriptine improves impaired circadian gh secretion in obese premenopausal women. J Clin Endocrinol Metab .

[2] Kok P, Roelfsema F, Frölich M, van Pelt J, Meinders AE, et al. (2006) Activation of dopamine d2 receptors lowers circadian leptin concentrations in obese women. J Clin Endocrinol Metab 91: 3236–3240.

[3] Kok P, Roelfsema F, Frolich M, van pelt J, Stokkel MPM, et al. (2006) Activation of dopamine d2 receptors simultaneously ameliorates various metabolic features of obese women. American Journal of Physiology- Endocrinology and Metabolism 291(5): 1038-1043.

[4] Vis DJ, Westerhuis JA, Hoefsloot HCJ, Pijl H, Roelfsema F, et al. (2009) Endocrine pulse identification using penalized methods and a minimum set of assumptions. Am J Physiol Endocrinol Metab .

[5] de la Fuente A, Bing N, Hoeschele I, Mendes P (2004) Discovery of meaningful associations in genomic data using partial correlation coefficients. Bioinformatics 20: 3565-3574.

[6] Box GEP (c1976) Time series analysis: forecasting and control. Holden-Day series in time series analysis and digital processing. San Francisco: Holden-Day, rev. ed. edition. Includes bibliographical references and indexes.

[7] Benjamini Y, Hochberg Y (1995) Controlling the false discovery rate: A practical and powerful approach to multiple testing. Journal of the Royal Statistical Society Series B (Methodological) 57: pp. 289-300.

[8] Fisher RA (1935) The Design of Experiments. UK: Edinburgh: Oliver and Boyd.

[9] Granger CWJ (1969) Investigating causal relations by econometric models and cross-spectral methods. Econometrica 37: 424-38.

[10] Pearl J (2000) Causality: models, reasoning, and inference. Cambridge, U.K: Cambridge University Press. URL http://www.loc.gov/catdir/description/cam0210/99042108.html.

[11] Pearl J (1995) Causal diagrams for empirical research. Biometrika 82: 669-688.

[12] Pearl J (2000) Comment: Causal thinking without counterfactuals. Journal of the American Statistical Association 95: 428-431.

[13] Dawid A (2004) Probability, causality and the empirical world: A bayes - de finetti - popper - borel synthesis. Statistical Science 19: 44.
